# Experimental investigation on the physical and mechanical properties enhancement of red clay modified with “RoadyesTM”

**DOI:** 10.1371/journal.pone.0333092

**Published:** 2025-10-17

**Authors:** Quan Shen, Yuhang Lv, Gai Liu, Liuyiyi Yang, Zhaohui Wang

**Affiliations:** 1 School of Civil Engineering, Hunan University of Technology, Zhuzhou Hunan, China; 2 Hunan provincial communications planning, survry & design institute, Changsha, China; Mirpur University of Science and Technology, PAKISTAN

## Abstract

This study investigates the enhancement of the physical and mechanical properties of red clay from the Zhuzhou region using a nano polymer material (RoadyesTM), cement, and lime. The effects of varying dosages of RoadyesTM, cement, and lime on the properties of the modified red clay are examined. The indicate reveal that: (1) the addition of RoadyesTM alone results in a reduction in both the liquid limit and plasticity index, while simultaneously increasing cohesion and unconfined compressive strength; (2) Red clay treated with 0.25% RoadyesTM demonstrates the highest compression index, indicating optimal compressibility.; (3) The combination of RoadyesTM with cement and lime significantly enhances the mechanical properties of the red clay. Specifically, the combinations of 0.25% RoadyesTM with 6% cement and 0.25% RoadyesTM with 7% lime lead to the most significant improvements in both cohesion and compressive strength, with only minor changes in the internal friction angle. Additionally, it is found that the optimal dosage of cement decreases by 9% when RoadyesTM is incorporated.; (4) The effectiveness of the combination of 0.25% RoadyesTM and 6% cement is notably superior to that of the combination with 7% lime. Overall, the results of this provide offer valuable insights for the design and construction of roadbed engineering involving red clay.

## 1. Introduction

The Red clay is widely distributed in the southern part of China. Red clay is formed through initial weathering, secondary weathering and later reconsolidation. In engineering applications, red clay, as a distinct soil type, exhibits issues such as poor geotechnical properties and inadequate drainage capacity. According to the Technical Specification for Highway Roadbed Construction” [1], red clay must be improved by blending and construction treatment to meet the specification requirements before being used as roadbed fill. In order to improve the engineering performance of red clay, numerous researchers have conducted in-depth research and practical studies. Zhou Daihui et al [2] demonstrated that cement and lime could reduce the liquid limit and plasticity index of red clay. Hu Wenhua et al [3] used cement and lime to improve red clay, and concluded that the CBR and unconfined compressive strength of improved red clay were larger when the amount of cement was 10%−15% or the amount of lime was 5%−10%. Liu Baochen et al [4] used cement mixing method to conduct indoor experimental research on the relationship between the unconfined compressive strength of Guilin soft red clay and the cement mixing ratio and other major mechanical properties, and concluded that the unconfined compressive strength of cemented red clay increases with the increase of the cement mixing ratio, the cement mixing ratio increases from 7% to 20% strength enhancement is obvious, and when the cement mixing ratio is greater than 20%, the strength growth tends to be slow. Zhang Hong et al [5] found that cement can improve the water sensitivity of red clay and inhibit shrinkage cracking. Liu Wu [6], Xiao Qingyi et al [7] found that lime can improve the bearing ratio and compressive strength of red clay to meet the requirements as a roadbed fill. Zeng Jun [8] obtained that lime can improve the compaction characteristics and compressive strength of red clay through indoor tests, and concluded that the optimum lime dosage is 8%.Tang et al [9] modified red clay by adding lime, fly ash and their mixtures. The results showed that lime and fly ash reduced the liquid limit, plasticity index and maximum dry density of red clay, increased the shear strength and unconfined compressive strength, and reduced the compressibility.Gao et al [10] found that the addition of 3% waste tire rubber powder could significantly improve the mechanical properties and structural characteristics of red clay, and the mechanical properties were enhanced by triaxial compression and piezomercury method tests.Wang et al [11] used lateral limit compression and straight shear tests to test the improvement effect of xanthan gum on the physical and mechanical properties of red clay. It was shown that 1.0% ~ 1.5% xanthan gum could effectively limit the compression deformation of red clay and improve its cohesion at the same time.Weng et al. [12] added polypropylene fibers on the basis of xanthan gum to modify red clay, and through the compressive strength test, it was found that xanthan gum and polypropylene fibers had a synergistic curing effect, which could improve the compressive strength of red clay more effectively.Song et al. [13] found that xanthan gum and polypropylene fibers had synergistic curing effect, which could improve the compressive strength of red clay more effectively through the straight shear test and the compressive strength test. Test and compressive strength test found that basalt fibers improved the cohesion and compressive strength of red clay to a certain extent, but the improvement was small. Wang Jiaquan et al [14] used sodium polyacrylate to modify red clay. The test results showed that the mechanical properties such as cohesion and disintegration resistance of modified red clay were significantly improved. However, at high dosage of fly ash, the modified clay would be brittle to a certain extent; at high dosage of lime and cement, the cost increased significantly and was not environmentally friendly enough. Therefore, this paper attempts to introduce a new curing agent to improve the hydrophilic properties of red clay, so as to reduce the dosage of cement and lime.

The Institute of Soil Science, Chinese Academy of Sciences has developed a new water-resistant and strong-based environmentally friendly curing agent called Roadyes [15], which is capable of permanently curing soil through polymer particle technology. This new material can not only improve the mechanical properties of soil, but also improve the hydrophilic properties of soil and inhibit the development of cracks. Li Hongru et al [16] studied the mechanical properties of modified loess using Roadyes combined with lime and cement. The results showed that the compression index and resilience index of the Roadyes combined with lime and cement modified loess decreased significantly, and the compression resistance of loess increased, among which 0.25% Roadyes combined with 3% cement had the best modification effect. And the effect of Roadyes combined with cement modified loess is better than Roadyes combined with lime modified loess. Zhao He [17] studied the mechanical properties of loess modified by Roadyes under the action of freeze-thaw cycle, and the results showed that after several freeze-thaw cycles, the shear strength, compression characteristics and other mechanical properties of the Roadyes modified soil were better than the plain loess. Zhu Haoxuan [18] found that the synergistic effect of fly ash-Roadyes can improve the disintegration characteristics of loess. Zhang Ming et al [19] combined cement, lime and Roadyes together to treat a highway project abandoned soil, the test found that the compressive strength and CBR value of the improved roadbed are greatly improved, environmental protection and high efficiency. This new curing agent provides a promising approach for improving the physical and mechanical properties of red clay. However, no reports have yet been found on the physical and mechanical properties of red clay modified by Roadyes. Therefore, this paper researches the red clay roadbed disease in Zhuzhou area, and intends to use the indoor geotechnical test method to study the physical and mechanical properties of the Roadyes with cement and lime to improve the red clay and its influence on the law of change, and to determine the optimal ratio for remediation or mitigation of red clay roadbed disease. The research results are expected to provide effective reference for the engineering improvement of red clay soil.

## 2. Research on engineering diseases of Zhuzhou red clay

**Fig 1:**
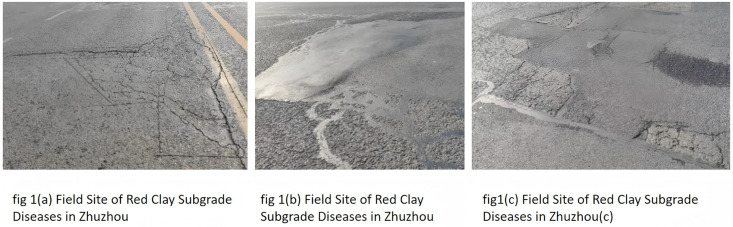
Field Site of Red Clay Subgrade Diseases in Zhuzhou.

According to the research on the disease of red clay roadbed in Zhuzhou area,common issues and pathologies have been identified in these roadbeds. The red clay roadbed of Hengshan West Road in Zhuzhou City exhibits a series of problematic phenomena in the pavement structure, as illustrated in Fig 1.There are the following reasons: (1) the red clay roadbed exhibits weak expansion. After absorbing moisture to produce a weak expansion of the roadbed to produce bulging deformation, due to the uneven bulging deformation, resulting in small cracks in the pavement; (2) construction and other factors. The red clay soil is not sufficiently compacted, and under the action of traveling load, uneven settlement occurs, which leads to pavement cracking; (3) the influence of groundwater. In the case of high groundwater level, the water content of red clay increases, the bearing capacity of the roadbed decreases, and the traveling load leads to pavement cracking. Due to the existence of pavement cracks, rainwater can enter the interior of the roadbed through the cracks. Under the action of dry and wet cycle in the atmosphere and the repeated crushing of traffic loads, it leads to roadbed subsidence, folds, slurry mud and other pathologies, which seriously threaten the comfort and safety of highway traffic. In this paper, we try to use Roadyes, cement, lime to improve the engineering properties of red clay, to improve or reduce the red clay roadbed subsidence, folds, slurry mud and other pathologies.

## 3. Overview of the test

### 3.1. Test material

**Fig 2:**
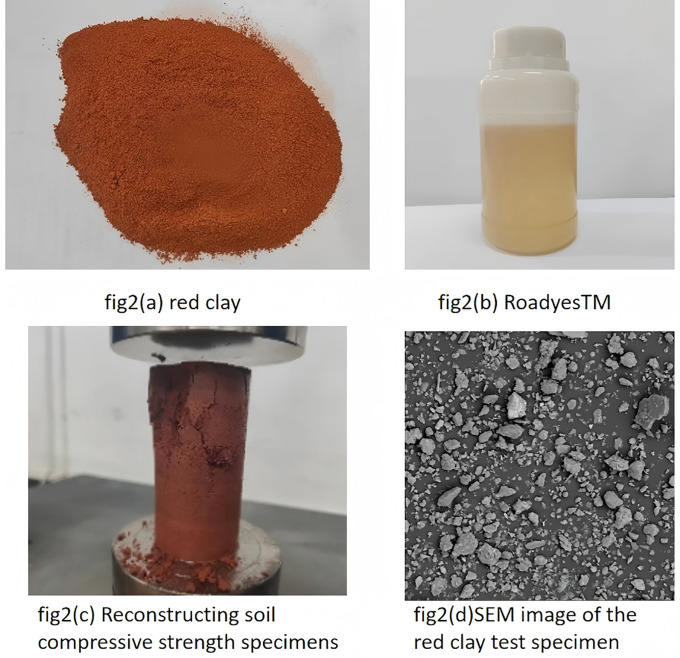
Test specimen.

**Fig 3:**
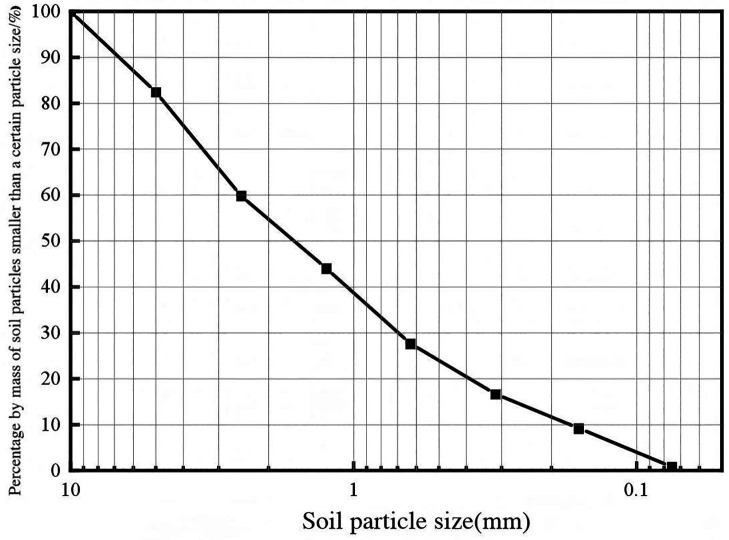
Particle size distribution curve.

“Roadyes”is a dark brown liquid with oily characteristics.Its main components include vinyl acetate-ethylene copolymer and spherical Al_2_O_3_ particles, which have a particle size ranging from 50 to 100 nm. When mixed with water, it appears white. This study selects red clay as the sample, which was collected from the landslide area near the embankment slope of Hengshan West Road in Zhuzhou. The soil is uniform, with fine particle size and a reddish-brown color. Due to the small quantity of the sample taken for the experiment and its location outside agricultural land, no additional permits are required at this stage. The soil sample was dried, crushed and sieved after retrieval. The physical and mechanical properties of the red clay were determined according to the geotechnical test method standard [20]. For the specific parameters, please refer to Table 1. The characteristics and particle size distribution of red clay are presented in Figs 2 and 3. The gradation of the red clay is shown in Fig 3.The test was conducted by using Roadyes, ordinary silicate cement and quicklime mixed with red clay according to a certain proportion to prepare samples.

**Table 1:** Physical and Mechanical Properties of Red Clay.

plastic limit(%)	liquid limit(%)	plasticity index Ip (%)	maximum dry density(g/cm3)	optimum soil moisture(%)	cohesion(kPa)	internal friction angle of soil(°)
25.8	52.9	27.1	1.63	20	25.8	10.16

### 3.2. Test methods

All the specimens used in this study were remolded, with soil samples prepared to have a natural moisture content of 17% and a dry density of 1.56, as illustrated in Fig 2. The proportions of Roadyes, cement, and lime admixtures were calculated based on their mass percentage relative to the dry soil, as defined in [21]. The Roadyes dosage was set at 0.25% and 0.5%, cement dosage was set at 3%, 6% and 9% and lime dosage was set at 4%, 7% and 10%. In carrying out the Roadyes amended soil test, the required Roadyes was mixed thoroughly with water and then sprayed evenly into the soil samples using a spray bottle. The details of the test procedure are presented in Table 2.

**Table 2:** Experimental Design.

serial number	Percentage of Roadyes mixing/%	Percentage of cement mixing/%	Percentage of lime mixing/%	Liquid Limit and Plastic Limit Tests	Direct shear test	Unconfined compression strength test	Consolidation test
1	0	–	–	Yes	Yes	Yes	Yes
2	0.25	–	–				
3	0.50	–	–				
4	0.25	3	–	–			
5	0.25	6	–	–			
6	0.25	9	–	–			
7	0.25	–	4	–			
8	0.25	–	7	–			
9	0.25	–	10	–			

#### 3.2.1. Atterberg limits test.

A certain amount of dried soil samples was passed through a 2 mm sieve, then mixed with Roadyes at mass fractions of 0.25% and 0.5%. The resulting solution was evenly sprayed onto the soil samples using a spray bottle. The joint liquid-plastic limit test was subsequently conducted in accordance with the geotechnical test methods s +tandard [20].

#### 3.2.2. Direct shear test.

A strain-controlled direct shear apparatus was employed to investigate the effects of different Roadyes mixes, including Roadyes-cement and Roadyes-lime combinations, on the cohesion and internal friction angle of red clay. A total of 9 test groups were conducted, each consisting of 4 specimens. The dimensions of the specimens were 2.0 cm in height (H) and 6.18 cm in diameter (D). The specimens were prepared according to the standard formulation and tested after a 7-day curing period.

#### 3.2.3. Unconfined compression strength test.

An electronic universal testing machine was employed to determine the compressive strength of Roadyes, Roadyes-cement, and Roadyes-lime combinations in improved red clay. A total of 9 test groups were conducted, with 3 specimens in each group. The dimensions of the specimens were 80 mm in height (H) and 39.1 mm in diameter (D). The tests were performed after a 7-day curing period.

#### 3.2.4. Consolidation test.

A total of nine test sets, each consisting of two specimens, were conducted using a triaxial consolidator to investigate the compression characteristics of Roadyes, Roadyes-cement, and Roadyes-lime combinations in improved red clay. The specimens had dimensions of 2.0 cm in height (H) and 6.18 cm in diameter (D) and were tested after a 7-day curing period.(The curing time refers to the consolidation time.)

## 4. Analysis of test results

### 4.1. Boundary moisture content indicators

**Fig 4:**
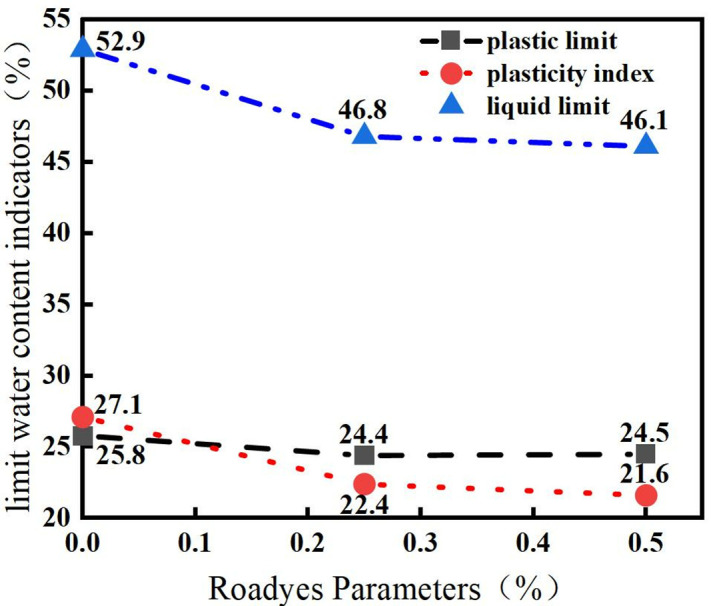
Roadyes-modified red clay water content limits.

The bounding moisture content index of the improved red clay soil is shown in Fig 4.As depicted in the figure,the liquid limit and plasticity index of the red clay soil modified with Roadyes decreased,This can be attributed to the ability of Roadyes to distribute water more evenly throughout the soil, reducing the adsorption capacity of the soil particles for water. As a result, both the liquid limit and plasticity index of the red clay soil decrease,altering the characteristics of the red clay, which typically exhibits high liquid limit and plasticity.According to the “Geotechnical Engineering Investigation Specification” [22],the high-plasticity clay, which is brownish-red or brownish-yellow in color and associated with the carbonate rock system, and whose liquid limit is greater than or equal to 50%, should be classified as primary red clay. The water content of the modified soil is 46.8% or 46.1%, both of which are lower than 50%. Therefore, the Roadyes-amended red clay can be classified as ordinary clay, thereby improving the physical properties of the red clay.

### 4.2. Direct shear test

**Fig 5:**
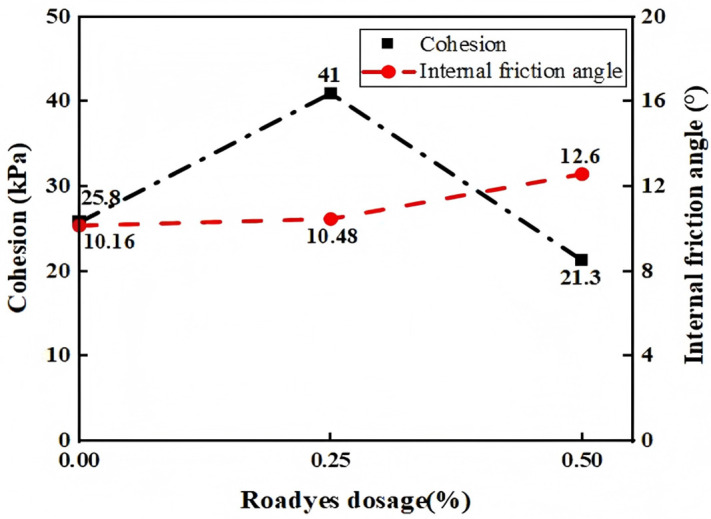
Influence of Roadyes on red clay shear strength.

**Fig 6:**
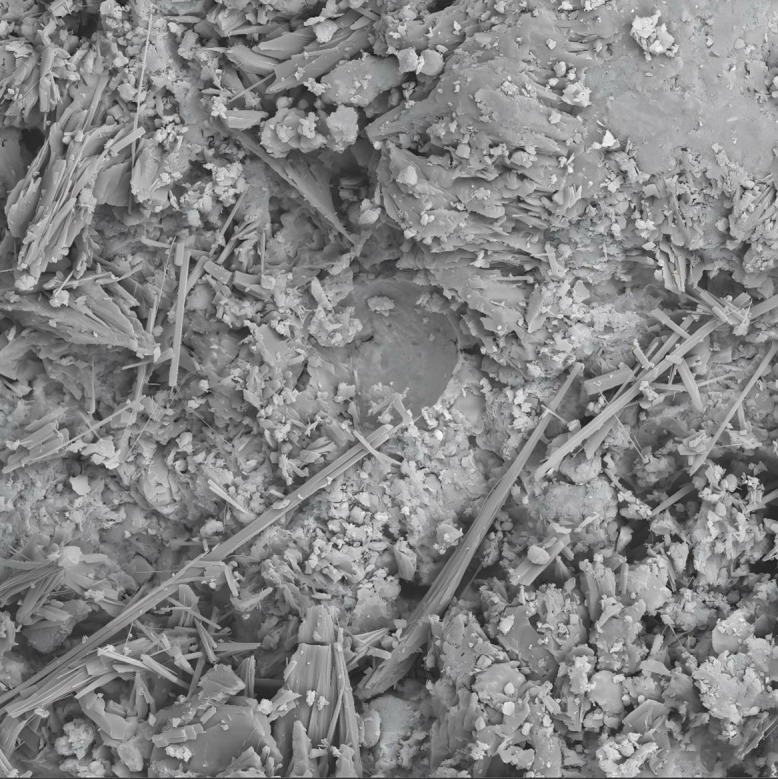
EMI of 0.25% Roadyes-treated red clay after failure.

The effect of the road liquid dosage on the shear strength parameters of red clay is shown in Fig 5. As observed from the figure, the cohesive strength of pure red clay is 25.8 kPa, and the internal friction angle is 10.16°. After the road liquid modification, the cohesive strength first increases and then decreases. When the road liquid dosage is 0.25%, the cohesive strength reaches its maximum value of 41 kPa, while the internal friction angle shows only a small variation. This is because, when 0.25% road liquid is added, Al_2_O_3_ in the red clay reacts with the road liquid to form Al_2_O_3_·3H_2_O gel. As shown in Fig 6 (electron microscope image), this gel acts as a binder for the soil particles, thereby increasing the cohesive strength. However, when 0.5% road liquid is added, the volume of the gel becomes too large, reducing the relative volume occupied by the soil particles. Under compression, excessive water and gas in the pores are not easily expelled, resulting in a decrease in cohesive strength.

**Fig 7:**
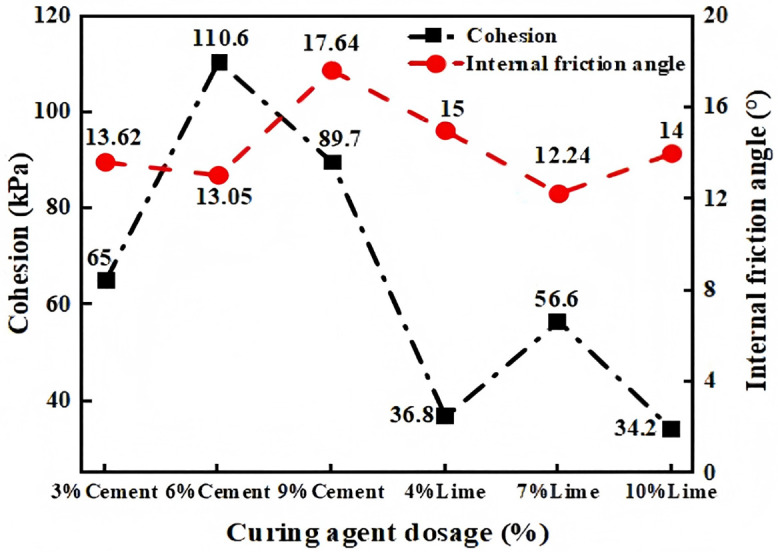
Effect of three substances on clay shear strength.

The improvement effects of 0.25% and 0.5% Roadyes dosing on the liquid limit and plasticity index of red clay are similar. However, 0.25% Roadyes dosing demonstrates a more significant enhancement in the cohesion of red clay. Therefore, we chose to combine 0.25% Roadyes with cement and lime, respectively, to evaluate the combined improvement effect. The influence of different dosages of cement and lime on the shear strength index of red clay is shown in Fig 7. As illustrated, the cohesive strength of the improved red clay increased by 152%, 329%, and 248% when the cement dosages were 3%, 6%, and 9%, respectively. In contrast, the cohesive strength increased by 43%, 119%, and 33% when the lime dosages were 4%, 7%, and 10%, respectively. The variation in the internal friction angle was minimal. The highest increase in cohesive strength occurred when the cement and lime dosages were 6% and 7%, respectively, suggesting that these are the optimal dosages for cement and lime. This improvement can be attributed to the formation of Ca(OH)₂ through the reaction of cement and lime, as well as the creation of CaO-SiO₂-H₂O and Al₂O₃-3H₂O gels from the interaction between Roadyes and the SiO₂ and Al₂O₃ components in the soil. These reactions help cement soil particles, strengthening their connections and filling soil pores, which ultimately enhances the cohesive force within the red clay. Consequently, the addition of cement and lime effectively promotes the enhancement of red clay cohesion in the presence of Roadyes.

### 4.3. Unconfined compression strength test

**Fig 8:**
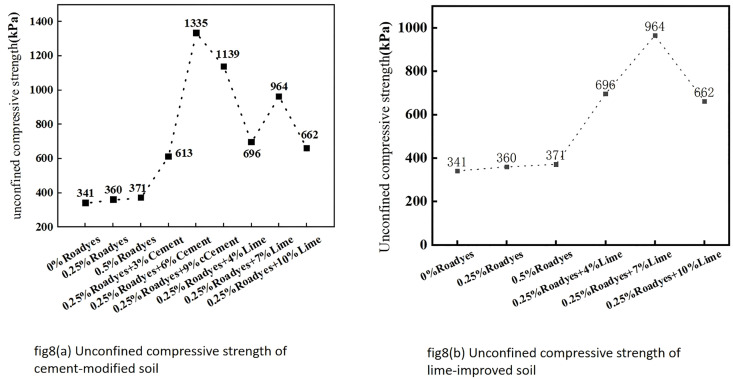
Unconfined Compressive Strength of Improved Red Clay.

The unconfined compressive strength of the modified red clay is presented in Fig 8. From the figure, it can be observed that the compressive strength of the improved red clay increases to varying extents with the addition of curing agents. Although the compressive strength of the Roadyes-amended red clay increased by only 9%, which represents a relatively small improvement, the compressive strength of the soil was significantly enhanced when Roadyes was combined with cement and lime. The compressive strength increased by 80%, 291%, and 234% when the cement content was 3%, 6%, and 9%, respectively. Similarly, the compressive strength increased by 104%, 183%, and 94% when the lime content was 4%, 7%, and 10%, respectively. Among these, the optimal cement and lime dosages were found to be 6% and 7%, respectively. The mechanism by which Roadyes, in combination with cement and lime, enhances the unconfined compressive strength of red clay is discussed in Section 4.2.

However, the compressive strength gradually decreases with the further increase of cement and lime dosage because, when the dosage in the dosage of cement and lime. This is because, when the amount of cement or lime is excessive, there may not be sufficient water in the soil to fully participate in the reaction, resulting in incomplete reaction of some curing agents or ineffective excess curing agents, which affects the saturated bonding strength of the soil particles. Additionally, an excess of lime can render the soil too alkaline, inhibiting the hydration reactions of certain minerals, thereby weakening the cohesion and overall strength of the soil. The combination of 0.25% Roadyes-6% cement and 0.25% Roadyes-7% lime resulted in the most significant improvement in cohesion and compressive strength of the red clay soil, with a relatively smaller change in the angle of internal friction. A comparison with the literature [3] reveals that the optimum dosage of cement was reduced by 4% after the incorporation of Roadyes. Similarly, a comparison with the literature [8] indicates that the optimum dosage of lime was reduced by 1% after the addition of Roadyes.

### 4.4. Consolidation test

#### 4.4.1. Void ratio and compression index.

**Fig 9:**
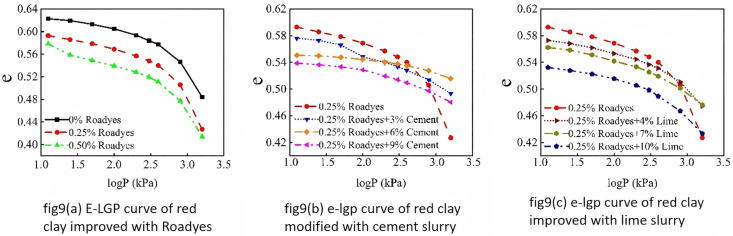
*e-lgp* curve of improved red clay.

**Fig 10:**
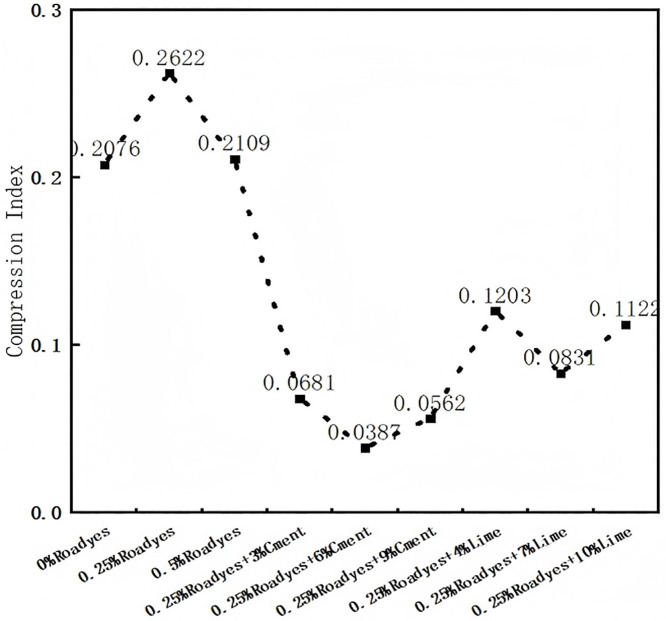
Compression index of improved red clay.

The Void ratio of modified red clay is illustrated in the figure below. As shown in Fig 9, the void ratio of red clay decreases with increasing load, and this decrease is influenced by the proportions of Roadyes,cement,and lime used.From Fig 9(a), it can be observed that the void ratio of red clay decreases as the amount of Roadyes admixture increases. When considering Fig 10,the compression index initially increases and then decreases under conditions where only Roadyes is added. The compression index of red clay with 0.25% Roadyes admixture is the highest, indicating the best compressibility. This suggests that 0.25% of Roadyes can significantly improve the compaction of the red clay roadbed, reduce the void ratio, and lower the hygroscopicity and permeability of the soil. Consequently, the physical properties of the red clay are enhanced, which in turn improves the bearing capacity of the roadbed. This improvement can be attributed to the reaction of Al_2_O_3_ in the soil with Roadyes and water, forming Al_2_O_3_-3H_2_O gel, which functions as a lubricant during the compaction process. This reduces friction between soil particles, making the soil more easily compacted. However, when 0.5% Roadyes is mixed into the soil, the gel volume becomes too large, and the relative volume occupied by the soil particles becomes small. As a result, excessive water and gas in the pores are not easily expelled during compaction, hindering the movement of soil particles and making the soil more difficult to compact. Therefore, the optimal admixture ratio is 0.25% Roadyes.

As shown in Figs 9(b) and (c), the reduction in the void ratio of red clay significantly decreases after the addition of cement and lime, resulting in a notable increase in the compressive deformation resistance of the improved soil. From Fig 10, it can be observed that the compression index first decreases and then increases when Roadyes-cement is added. The compression index reaches its minimum value when 0.25% Roadyes and 6% cement are added, showing an 81% decrease. Similarly, for the Roadyes-lime mixture, the compression index initially decreases and then increases, with the smallest value occurring when 0.25% Roadyes and 7% lime are added, resulting in a 58% decrease. This improvement is attributed to the addition of the curing agents, which generate a gel that fills the pores within the soil, increasing its density and, consequently, enhancing the compressive deformation resistance of the soil.

#### 4.4.2. Lateral compression strain.

**Fig 11:**
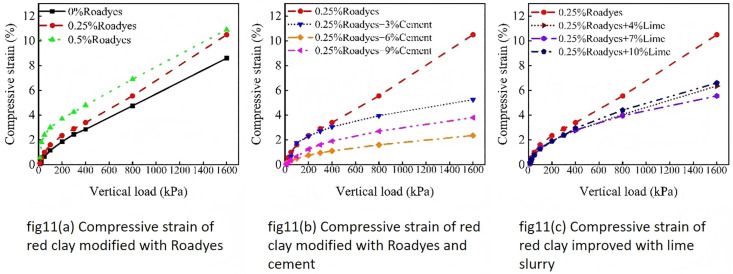
Compressive strain of improved red clay.

The compressive strain of the improved red clay is illustrated in Fig 11. As shown in Fig 11(a), the compressive strain of the red clay soil increased after treatment with Roadyes, indicating that Roadyes enhances the compressibility of the red clay soil. As shown in Fig 11(b) and (c), the soil became hardened and the compressive strain was significantly reduced after the incorporation of cement and lime, suggesting that the addition of cement and lime substantially improves the compressive capacity of the red clay soil.

**Fig 12:**
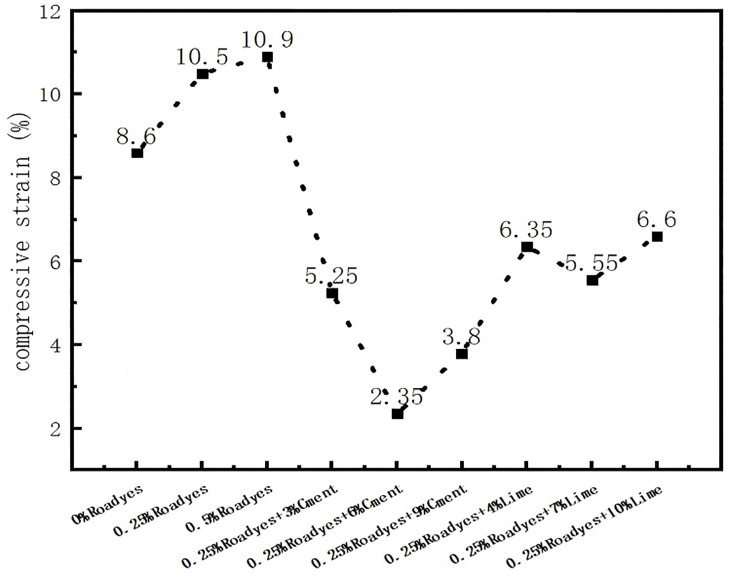
Compressive strain of improved red clay at 1600 kPa.

The compressive strain of modified red clay under 1600 kPa load is shown in Fig 12. As observed in the figure, the compressive strain of the red clay increases when only Roadyes is mixed, which can be attributed to the formation of an Al_2_O_3_-3H_2_O gel by the action of Roadyes. This gel reduces the friction between soil particles, enhancing the compressibility of the red clay, making it easier to compress, thus resulting in an increase in compressive strain. However, when cement and lime are mixed, the compressive strain of the red clay is effectively reduced. Under the condition of Roadyes-cement, the compressive strain first decreases and then increases, with the smallest compressive strain observed when 0.25% Roadyes and 6% cement are mixed, showing a reduction of 72%. Under the condition of Roadyes-lime, the compressive strain also decreases and then increases, with the smallest compressive strain occurring when 0.25% Roadyes and 7% lime are mixed, showing a reduction of 35%. This indicates that the improved soil exhibits high strength, with minimal deformation under high load pressure. Compared to lime, the synergistic combination of Roadyes and cement yields a better improvement effect. The mechanism through which Roadyes-cement and Roadyes-lime improve the deformation resistance of red clay is consistent with that discussed in Section 4.4.1.

#### 4.4.3. compressibility modulus.

**Fig 13:**
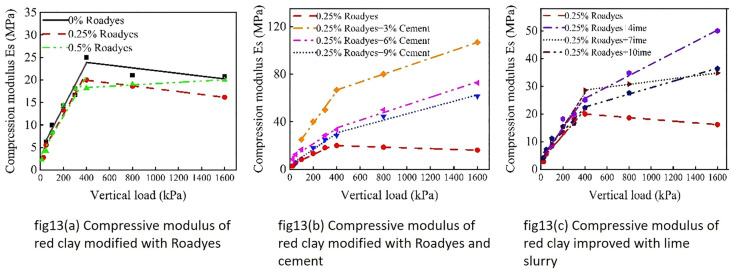
Compressibility modulus of improved red clay.

The compressive modulus of the modified red clay was fitted in two segments due to the differing growth trends of the compressive modulus under varying vertical loads. The straight-line fitting of the compression modulus for the modified red clay is shown in Fig 13. As illustrated in Fig 13(a), the compressive modulus of the improved red clay increased linearly until the vertical load reached 400 kPa. Beyond this point, when the vertical load increased to 400 kPa, the compressive modulus of the Roadyes-modified red clay exhibited a decreasing trend, while the compressive modulus of the 0.5% Roadyes-modified soil continued to increase slightly. This behavior is attributed to the formation of Al_2_O_3_-3H2O gel, which facilitates the compression of the soil. When the vertical load exceeds 400 kPa, the strain growth in the soil becomes more pronounced, resulting in a decrease in the compressive modulus. As shown in Fig 13(b) and (c), before reaching a vertical load of 400 kPa, the compressive modulus of the modified red clay soil increased linearly, with a faster growth rate. However, once the vertical load surpassed 400 kPa, the growth rate of the compressive modulus for the Roadyes-modified red clay mixed with cement and lime slowed down. This is because, as the load reaches a certain threshold, the soil structure becomes more compact, and further increases in vertical load lead to a reduced rate of compression deformation, which causes the compressive modulus to grow at a slower rate.

**Fig 14:**
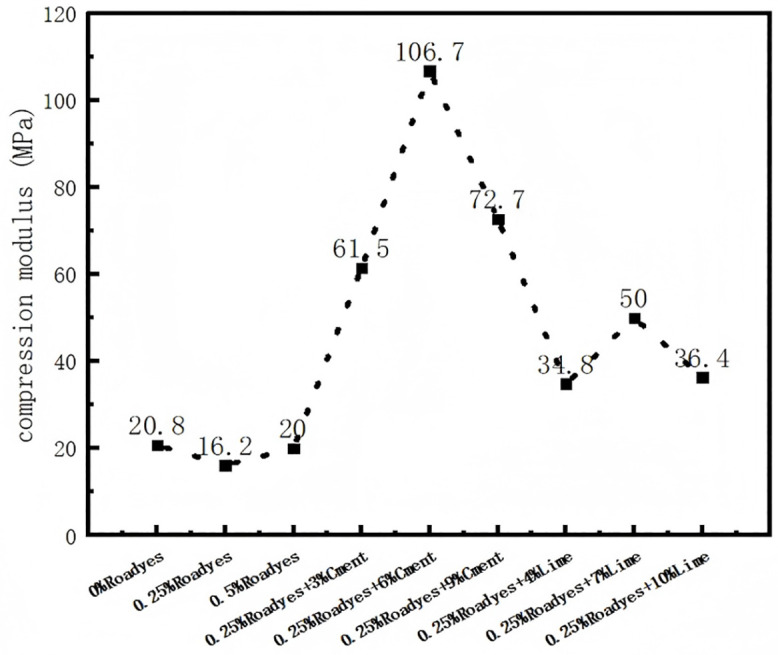
Compression modulus of improved red clay at 1600 kPa.

The compression modulus of the improved red clay soil under a 1600 kPa load is shown in Fig 14. As observed in the figure, the compressive modulus of the soil decreased after modification with Roadyes. This reduction is attributed to the Al_2_O_3_-3H_2_O gel generated by the reaction, which reduced the friction between soil particles, making the soil more easily compressed and thus decreasing the compressive modulus. However, after mixing with cement and lime, the compressive modulus of the improved soil increased significantly, exhibiting a trend of first increasing and then decreasing. The compressive modulus of the red clay soil was the highest when 0.25% Roadyes and 7% lime were mixed, with an increase of 181%. The maximum compressive modulus was observed when 0.25% Roadyes and 6% cement were mixed, showing an increase of 519%. When excessive cement and lime were added, the compressive modulus decreased, likely due to the rapid early-stage gelling reaction. This accelerated reaction formed a gel that encapsulated the remaining curing agents, thereby inhibiting further adequate reactions. The mechanism by which cement and lime enhance the compressive modulus of red clay is detailed in Section 4.5.

### 4.5. Roadyes, cement, lime curing mechanism

The chemical reaction mechanism of Roadyes, Roadyes-lime, and Roadyes-cement-amended red clay soil, as analyzed by Guo Xiaohan [21], can be described as follows: The vinyl acetate-ethylene copolymer (VAE) in Roadyes is an emulsion, and when moisture is present in the soil, the VAE molecules form a thin film on the surface of the soil particles. This thin film enhances the bonding force between the soil particles, thereby reducing the plasticity and water sensitivity of the soil. Furthermore, the VAE copolymers are adsorbed physically onto the mineral surface of the red clay, which improves the microstructure of the soil. Roadyes also contains nano-sized spherical Al_2_O_3_ particles, which react with the silicate and aluminate in the red clay to form compounds similar to geopolymers. This reaction contributes to the enhancement of the soil’s cohesion and the formation of a more stable solidified structure. Additionally, the Al_2_O_3_ particles fill the pores and cracks in the soil, which leads to a reduction in porosity, an increase in compactness, and an overall improvement in the mechanical properties of the soil.

After the addition of lime, the reaction between lime (CaO) and the moisture in the red clay generates Ca(OH)_2_. This reaction increases the pH of the soil, promoting ion exchange reactions within the clay minerals. The Ca^2^ ⁺ ions replace monovalent ions such as Na⁺ and K⁺ in the clay minerals, leading to flocculation of the soil particles and a reduction in the soil’s plasticity and expansiveness.[23] Meanwhile, the generated Ca(OH)_2_ reacts with the silicates and aluminates in the red clay in a pozzolanic reaction, forming calcium silicate and calcium aluminate compounds, which play a cementing role and enhance the strength and stability of the soil. Furthermore, lime and Roadyes exhibit a synergistic effect: lime increases the pH of the soil, which facilitates the reaction of nano-Al_2_O_3_ particles with silicates and aluminates. [24] It also promotes the bonding effect of VAE copolymers with soil particles. The flocculation and cementation effects of lime, combined with the film formation and pore-filling properties of Roadyes, significantly improve the microscopic structure of the red clay, thereby enhancing the soil’s mechanical properties and durability.

Compared to the Roadyes-lime combination, the Roadyes-cement-modified soil exhibits smaller pores. This is due to the large quantities of 3CaO-SiO_2_ and 2CaO-SiO_2_ in the cement, which, through hydration reactions with water, generate hydrated calcium silicate (C-S-H), Ca(OH)_2_, and other products. These substances play a crucial role in the cementation of soil particles, significantly enhancing the bonding force between them, thereby improving the strength and stability of the soil. During cement hydration, the Ca(OH)_2_ produced reacts with the active silicates and aluminates in the red clay, triggering secondary reactions that form calcium silicate and calcium aluminate compounds, further enhancing the cementation and durability of the soil. Additionally, the Ca(OH)_2_ generated in the hydration process increases the pH of the soil, which facilitates the reaction of nano-Al_2_O_3_ particles with silicates and aluminates in the soil. This also aids in the formation of a stronger film on the surface of the soil particles by the VAE copolymers. The cement’s cementing effect, combined with the film formation and pore filling properties of Roadyes, results in a more stable and compact soil structure. The chemical reactions of the cement complement the physical and chemical actions of Roadyes, collectively enhancing the soil’s strength, stability, and water resistance.

## 5. Conclusion

(1) The Roadyes can make the water more evenly distributed in the soil, weaken the adsorption capacity of soil particles to water, thus reducing the liquid limit and plasticity index of red clay soil, and changing the characteristics of red clay soil with high liquid limit and high plasticity.
(2) The synergistic improvement effect of Roadyes, cement and lime on red clay is remarkable. In the combination of 0.25% Roadyes-6% cement and 0.25% Roadyes-7% lime, The compressive strength of red clay was optimized., the change of internal friction angle is small, and the improvement effect is stable and controllable.
(3) The red clay mixed with 0.25% Roadyes has the largest compression index and the best compressibility. Under the condition of 0.25% Roadyes-6% cement improvement, the red clay has the lowest compression index and compression strain, the highest compression modulus, and the strongest compression resistance.
(4) In the combination of Roadyes and cement, the optimal cement dosage is 6%; in the combination of Roadyes and lime, the optimal lime dosage is 7%, and the improved effect of Roadyes-cement combination is obviously better than that of Roadyes-lime combination; comparing with the improved red clay soil mixed with cement and lime only, the optimal dosage of cement was reduced by 4% and the optimal dosage of lime was reduced by 1% after adding Roadyes.
(5) The Roadyes formed a protective film on the surface of soil particles through vinyl acetate-ethylene copolymer (VAE), which enhanced the particle bonding and reduced the plasticity; the nano-sized Al_2_O_3_ particles further facilitated the formation of polymer and the filling of pore space, which improved the soil compactness. The addition of lime and cement raised the soil pH and promoted the reaction of nano-Al_2_O_3_ particles with silicate and aluminate in the soil, which combined with the thin film effect of the Roadyes to further improve the strength, stability and water resistance of the soil.

## Supporting information

S1 file Experimental data support. (XLSX)
